# Characterization of the complete chloroplast genome of *Actinidia melliana* (Actinidiaceae)

**DOI:** 10.1080/23802359.2025.2611483

**Published:** 2026-01-01

**Authors:** Tao Zhang, Yunzhi Lin, Yongsheng Liu, Yingzhen Wang

**Affiliations:** ^a^ School of Forestry Science and Technology, Lishui Vocational and Technical College, Lishui, China; ^b^ School of Horticulture, Anhui Agricultural University, Hefei, China

**Keywords:** *A. melliana*, chloroplast genome, phylogenetic analysis

## Abstract

*Actinidia melliana* is a unique *Actinidia* species whose genomic data is extremely lacking. In this study, we reported the complete chloroplast genome of *A. melliana* for the first time, which has a total length of 156,159 bp, consisting of a large single-copy (LSC) region of 90,525 bp, a small single-copy (SSC) region of 20,578 bp, and a pair of inverted repeat (IR) regions with 22,528 bp each. A total of 133 genes were annotated, including 84 protein-coding genes, 41 transfer RNA genes (tRNA) and 8 ribosomal RNA genes (rRNA). Phylogenetic analysis alongside 28 other species in the Actinidiaceae family revealed that *A. melliana* is most closely related to *A. cylindrica* var. *cylindrica*. This study provides new genetic resources for evolutionary studies of *Actinidia* and supports future efforts in species identification and conservation within the genus.

## Introduction

Kiwifruit, known for its exceptionally high vitamin C content, is an economically and nutritionally important fruit crop worldwide. The genus *Actinidia* exhibits rich genetic diversity (Huang et al. 2013), and the extensive morphological disparity in the genus’ vegetative and reproductive characters has consistently hindered its taxonomic classification (Li et al. 2007). Therefore, developing genomic resources for the *Actinidia* genus will facilitate its taxonomy, identification, conservation, and utilization. Besides the cultivated species such as *A. chinensis* and *A. deliciosa*, many wild *Actinidia* species existing in the wild remain unexploited.

**Figure 1.**
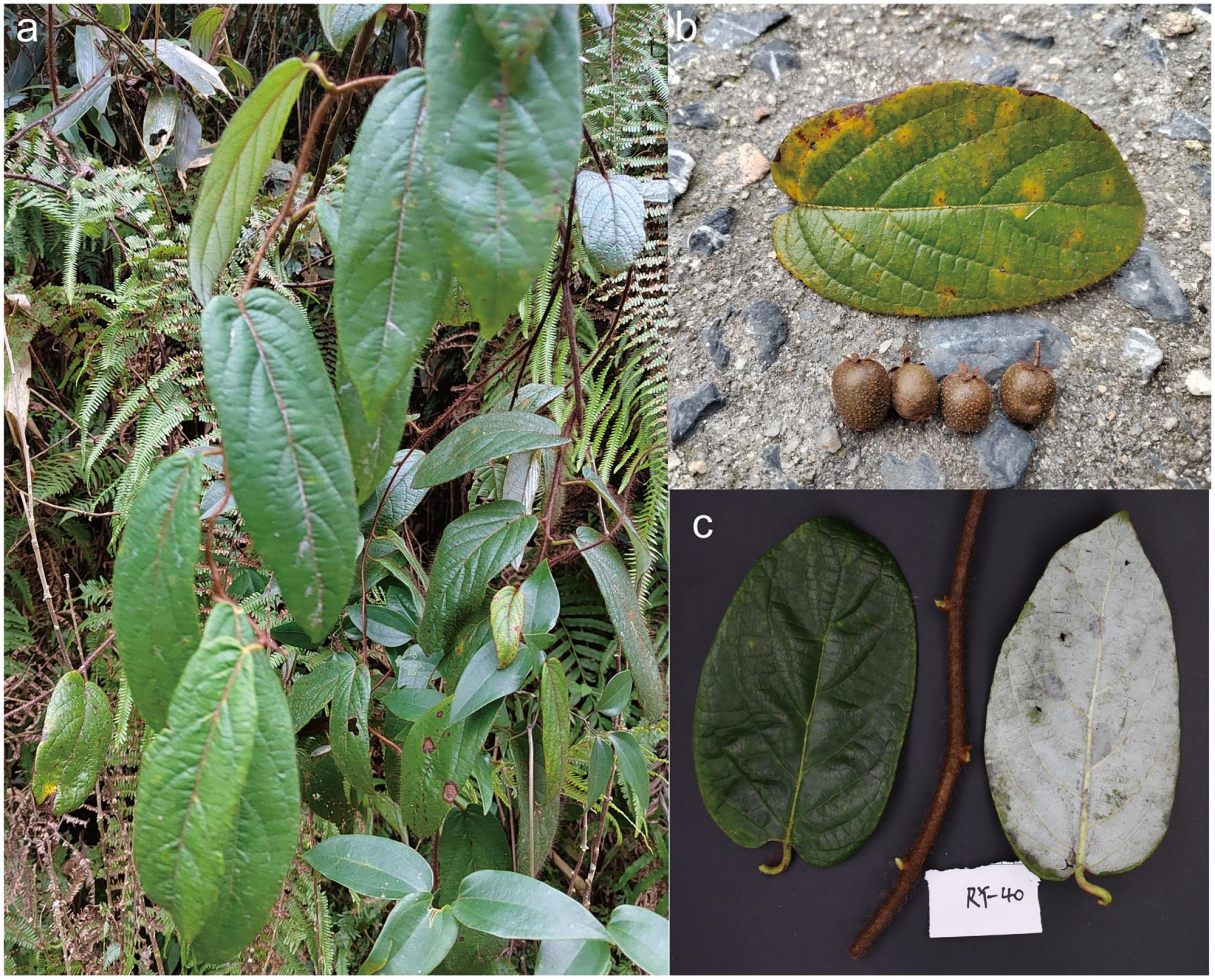
Morphology of *Actinidia melliana*. (a) Plant habit and surrounding vegetations; (b) leaf and fruits; (c) stem with characteristic brown and stiff hairs and leaves with the distinctive white abaxial surface (right). Samples were collected and photographed by Yingzhen Wang.

*Actinidia melliana* Hand.-Mazz., 1922 (Huang et al. 2013), a wild relative of cultivated kiwifruit, is primarily distributed in southern China, including the provinces of Guangxi, Guangdong, Hainan, Hunan, and Jiangxi (Huang et al. 2013). It typically grows in mountainous thickets at elevations between 200 and 800 meters. Compared to other kiwifruit species, *A. melliana* has a relatively restricted distribution, which contributes to its rarity in the wild. It is characterized by dense, brown and stiff hair on its stems and leaves, and a distinctly whitish underside of the leaves (Figure 1). However, due to a lack of genomic resources, its evolutionary relationships with other *Actinidia* species remain unclear. Chloroplast genome sequences are key genetic resources for revealing plant phylogeny and evolution, as well as for applications in species identification, classification, and conservation (Daniell et al. 2016). Therefore, resolving the evolutionary position of *A. melliana* through chloroplast genome assembly is both necessary and feasible.

In the present study, we sequenced and assembled the chloroplast genome of *A. melliana* using third-generation sequencing technology of the PacBio Sequel II platform for the first time. Through annotation and evolutionary analysis, this study initially reveals the structural features of the *A. melliana* chloroplast genome and its phylogenetic relationships with other *Actinidia* species, thereby contributing to the understanding of genetic diversity and evolutionary history within the genus.

## Materials and methods

### Plant material collection and DNA extraction

Leaf samples of *A. melliana* were collected from Ruyuan Yao Autonomous County, Guangdong Province, China (24.73°N, 113.07°E), in October 2021. The plant material was identified by Yongsheng Liu. The specimens were deposited at the Herbarium of Lishui Vocational & Technical College under specimen number RY-41 (contact person: Yingzhen Wang; 803296@lszjy.edu.cn). Total genomic DNA was extracted using a modified CTAB method (Allen et al. 2006) and evaluated using an Agilent 2100 Bioanalyzer (Agilent Technologies, Santa Clara, CA, USA) and Qubit fluorometer instrument (Thermo Fisher Scientific, MA, USA). A standard SMRTbell library was prepared with 50 μg of gDNA by using the SMRTbell Express Template Prep Kit 2.0, according to the manufacturer’s instructions. SMRTbell libraries were then sequenced on a PacBio Sequel II system (Pacific Biosciences, CA, USA).

### Sequence assembly and annotation

The sequencing output from the PacBio Sequel II system was processed using the SMRT Analysis software suite (v5.1.0) (https://www.pacb.com/products-and-services/analytical-software/smrt-analysis). The CCS subprogram (https://github.com/PacificBiosciences/ccs) was used with default settings to generate consensus HiFi reads sized 24.3 Gb in total. Oatk software (v1.0) (Zhou et al. 2025) was employed to assemble the complete chloroplast genome with parameter ‘-c 100 -p OatkDB/v20230210/angiosperm_pltd.fam’. The chloroplast genome of *A. melliana* was annotated and visualized using Geseq (Tillich et al. 2017), followed by manual adjustments according to the homologous genes of *A. lanceolata* (NC_046507.1) (Zhang et al. 2019). The annotated complete chloroplast genome was submitted to GenBank with the assigned accession number of PX646816.

### Phylogenetic analysis

To investigate the evolutionary history of *A. melliana*, we constructed a phylogenetic tree using 28 published chloroplast genomes from other Actinidiaceae species, with *Clematoclethra scandens* subsp. *hemsleyi* (derived from a genus within the family Actinidiaceae) (KX345299.1) (Wang et al. 2016) as the outgroup. Sequences were aligned with MAFFT (Nakamura et al. 2018) and trimmed with trimal (using -automated1 parameter) to construct a maximum-likelihood (ML) tree in RAxML (Stamatakis 2014) with 1000 bootstrap replicates. The evolutionary tree was finally visualized using the iTOL webtool (https://itol.embl.de/).

## Results

**Figure 2.**
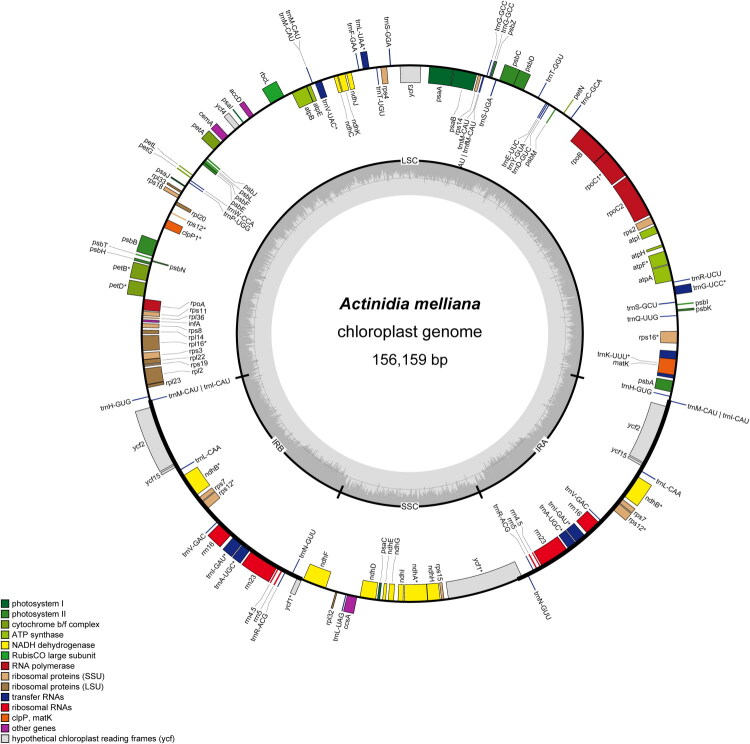
The complete chloroplast genome map of *A. melliana.* This map was generated using the OGDRAW (v1.3.1) tool on the Geseq platform and presents the annotation of 133 genes: 84 protein-coding genes, 41 tRNA genes, and 8 rRNA genes. Genes on the outer and inner rings are transcribed clockwise and counterclockwise, respectively. Functional groups are color-coded in the legend, and the inner gray circle shows the GC content.

The total length of the chloroplast genome of *A. melliana* is 156,159 bp (Figure 2). It exhibits a typical quadripartite structure, comprising one large single-copy region (LSC: 90,525 bp), one small single-copy region (SSC: 20,578 bp), and a pair of inverted repeat (IR) regions (IRA and IRB: 22,528 bp each). The total GC content was 37.3%, and that of the LSC, SSC, IRA, and IRB were 35.5%, 31.1%, 43.7%, and 43.7%, respectively. The coverage depth of the genome assembly was 4411.9× (Figure S1). A total of 133 genes were annotated, including 84 protein coding genes, 41 transfer RNA genes (tRNA) and 8 ribosomal RNA genes (rRNA). Among the 84 protein coding genes, 12 genes were identified as *cis*-splicing genes (Figure S2). Additionally, the rps12 gene is a *trans*-splicing gene with three unique exons (Figure S3).

**Figure 3.**
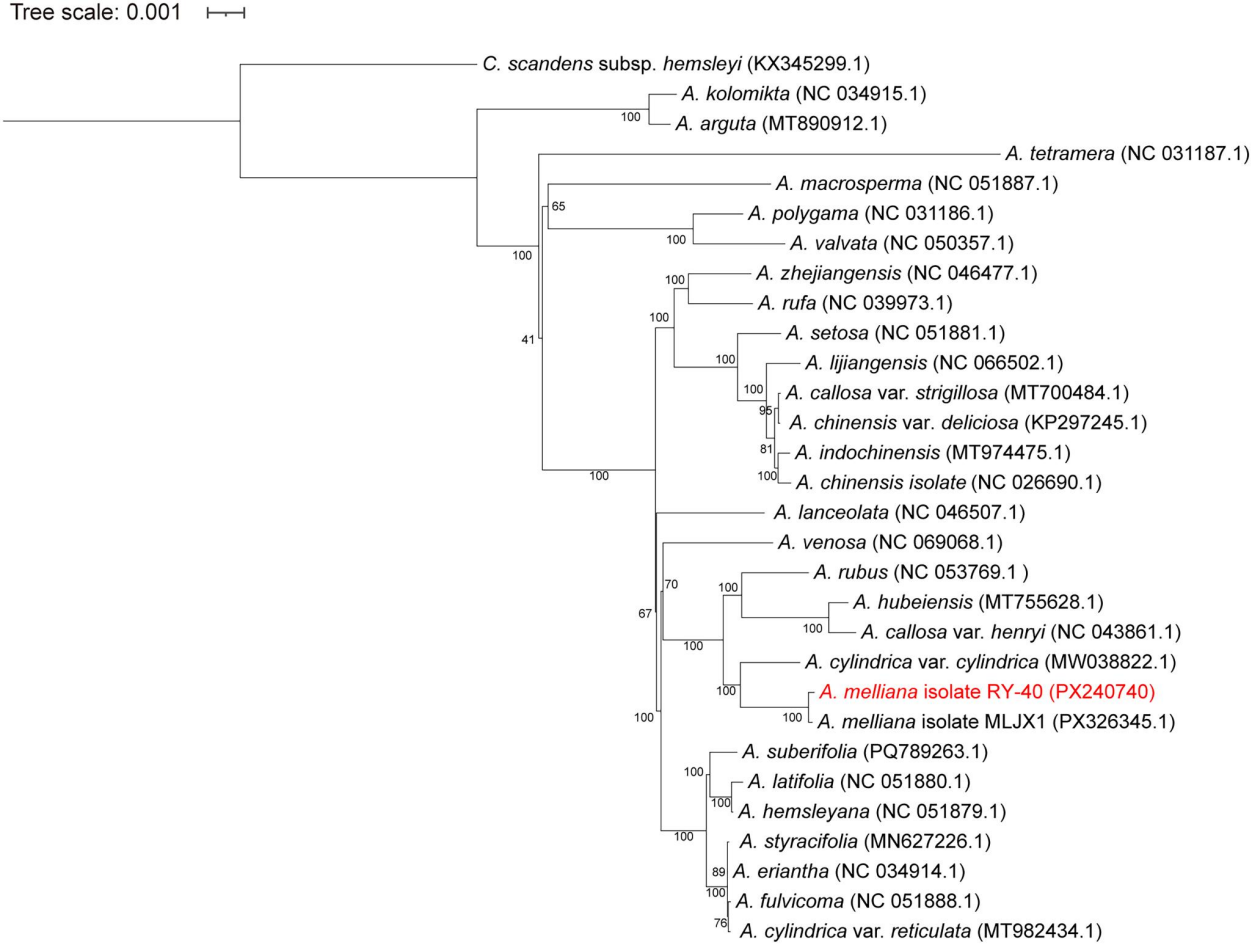
Maximum likelihood phylogenetic tree based on chloroplast genome sequences. The newly reported *A. melliana* is highlighted in red. *Clematoclethra scandens* subsp. *hemsleyi* (Wang et al. 2016) served as outgroup. The dataset included the following species: *A. kolomikta* (Lan et al. 2018), *A. arguta* (Ding et al. 2021), *A. tetramera* (Wang et al. 2016), *A. macrosperma* (Chen et al. 2019), *A. polygama* (Wang et al. 2016), *A. valvata* (Lin et al. 2020), *A. zhejiangensis* (Ai and Liu 2019), *A. rufa* (Kim et al. 2018), *A. setosa* (Lin et al. 2019), *A. lijiangensis* (Lin et al. 2025), *A. callosa* var. *strigillosa* (Liu et al. 2020), *A. chinensis* var. *deliciosa* (Yao et al. 2015), *A. indochinensis* (Lin et al. 2025), *A. chinensis* (Yao et al. 2015), *A. lanceolata* (Zhang and Liu 2019), *A. venosa* (Fu et al. 2023), *A. rubus* (Xu et al. 2020), *A. hubeiensis* (Lan et al. 2018), *A. callosa* var. *henryi* (Wu et al. 2019), *A. cylindrica* var. *cylindrica* (Ma and Liu 2019), *A. suberifolia* (Zhang et al. 2025), *A. latifolia* (Ren et al. 2023), *A. hemsleyana* (Qi et al. 2021), *A. styracifolia* (Yang et al. 2019), *A. eriantha* (Tang et al. 2019), *A. fulvicoma* (Zhang et al. 2019), and *A. cylindrica* var. *reticulata* (Lin et al. 2025).

We integrated the chloroplast genomes of 28 other *Actinidia* species and constructed a phylogenetic tree using *Clematoclethra scandens* subsp. *hemsleyi* as the outgroup to elucidate the evolutionary position of *A. melliana* (Figure 3). The results indicated that *A. melliana* is most closely related to *A. cylindrica* var. *cylindrica* (Ma and Liu 2019). Furthermore, species within the same clade as *A. melliana* included *A. rubus*, *A. hubeiensis*, and *A. callosa* var. *henryi*. This clade was fully supported with all branches receiving 100% bootstrap support value, indicating strong phylogenetic resolution for this clade.

## Discussion and conclusion

In this study, we reported the chloroplast genome of *A. melliana* for the first time, providing a valuable resource for investigating genetic diversity and evolutionary history within the family Actinidiaceae. The chloroplast genome of *A. melliana* has a total length of 156,159 bp and 37.3% GC content, which is consistent with the size range observed in other published *Actinidia* species (155,931 ∼ 157,639 bp; 37.0% ∼ 37.3%) (Lin et al. 2025).

Subsequent to the submission of our chloroplast genome, another genome assembly of *A. melliana* (GenBank: PX326345), collected from a distinct location in Hunan Province of China, became publicly available, which presents a unique opportunity for a comparative assessment. The two assemblies were generated using substantially different sequencing technologies: our study employed the third-generation long-read sequencing platform (Sequel II platform of PacBio), whereas PX326345 was based on the second-generation short-read sequencing platform (T7 platform of BGI). The chloroplast genome assembled in our study is 156,159 bp in length, 115 bp longer than that of PX326345, with the difference located in the LSC region. This discrepancy could be attributed to the different sequencing technologies employed, or it may represent genuine intraspecific variation between the individual samples. Importantly, phylogenetic analysis demonstrated that both sequences are closely clustered into a unique clade (Figure 3) and share 99.92% identity (Figure S4), strongly suggesting both sequenced samples/accessions were derived from an identical taxonomic unit.

Among the 28 published Actinidiaceae species, *A. melliana* is most closely related to *A. cylindrica* var. *cylindrica*. However, *A. melliana* contained one additional protein-coding gene, two additional tRNA genes, and one additional rRNA gene compared to *A. cylindrica* var. *cylindrica* (Ma and Liu 2019). This study provides molecular evidence for the evolutionary position of *A. melliana* for the first time, thereby supporting future efforts in the taxonomy, germplasm conservation, and utilization of *Actinidia* species.

## Supplementary Material

FigS3.tif

FigS4.tif

FigS1.jpg

figS2.tif

## Data Availability

The complete chloroplast genome sequence of *A. melliana* is available in the GenBank of NCBI under the accession number PX646816; the associated BioProject, Biosample, and SRA numbers are PRJNA1313282, SAMN50890997 and SRR35198340.

## References

[CIT0001] Allen GC, Flores-Vergara MA, Krasynanski S, Kumar S, Thompson WF. 2006. A modified protocol for rapid DNA isolation from plant tissues using cetyltrimethylammonium bromide. Nat Protoc. 1(5):2320–2325. 10.1038/nprot.2006.384 17406474

[CIT0002] Ai F, Liu H. 2019. The complete chloroplast genome sequence of *Actinidia zhejiangensis*. Mitochondrial DNA Part B. 4(1):690–691. 10.1080/23802359.2019.1573117

[CIT0003] Chen Y et al. 2019. The complete chloroplast genome of *Actinidia macrosperma*. Mitochondrial DNA B Resour. 4(2):4188–4189. 10.1080/23802359.2019.1692733 33366376 PMC7707781

[CIT0004] Daniell H, Lin CS, Yu M, Chang WJ. 2016. Chloroplast genomes: diversity, evolution, and applications in genetic engineering. Genome Biol. 17(1):134. 10.1186/s13059-016-1004-2 27339192 PMC4918201

[CIT0005] Ding F et al. 2021. The complete chloroplast genome sequence of *Actinidia arguta* var. *giraldii*. Mitochondrial DNA B Resour. 6(2):413–414. 10.1080/23802359.2020.1870884 33659696 PMC7872529

[CIT0006] Fu CN, Wicke S, Zhu AD, Li DZ, Gao LM. 2023. Distinctive plastome evolution in *carnivorous angiosperms*. BMC Plant Biol. 23(1):660. 10.1186/s12870-023-04682-1 38124058 PMC10731798

[CIT0007] Huang H et al. 2013. Actinidia: taxonomy, resources, domestication, cultivation. Science Press.

[CIT0008] Kim SC, Lee JW, Baek SH, Lee MW, Kang YJ. 2018. The complete chloroplast genome sequence of *Actinidia rufa* (Actinidiaceae). Mitochondrial DNA B Resour. 3(2):564–565. 10.1080/23802359.2018.1450676 33474242 PMC7799798

[CIT0009] Lan Y et al. 2018. The complete chloroplast genome sequence of *Actinidia kolomikta* from north China. Conservation Genet Resour. 10(3):475–477. 10.1007/s12686-017-0852-8

[CIT0010] Li JQ, Li XW, Soejarto DD. 2007. A revision of the genus *Actinidia* from China. Acta Hortic. 753(753):41–44. 10.17660/ActaHortic.2007.753.2

[CIT0011] Lin H, Xu Y, Bai D. 2020. The complete chloroplast genome of *Actinidia valvata* (Actinidiaceae). Mitochondrial DNA Part B. 5(2):1607–1608. 10.1080/23802359.2020.1745105 PMC7748799 33366559

[CIT0012] Lin H, Jiang L, Zhang F, Bai D. 2019. Assembly and phylogenetic analysis of the complete chloroplast genome sequence of *Actinidia setosa*. Mitochondrial DNA B Resour. 4(2):3679–3680. 10.1080/23802359.2019.1678423 33366140 PMC7707611

[CIT0014] Lin Q et al. 2025. Comparative chloroplast genomics provides insights into the phylogenetic relationships and evolutionary history for *Actinidia* species. Sci Rep. 15(1):13291. 10.1038/s41598-025-95789-y 40246989 PMC12006428

[CIT0015] Liu Y, Xie X, Qi X, Zhong C, Li D. 2020. Phylogenetic relationship and characterization of the complete chloroplast genome of *Actinidia callosa* var. *strigillosa*. Mitochondrial DNA Part B. 5(3):3420–3421. 10.1080/23802359.2020.1823258

[CIT0016] Ma X, Liu H. 2019. The complete chloroplast genome sequence of *Actinidia cylindrica* C. F. Liang. Mitochondrial DNA Part B. 4(1):1694–1695. 10.1080/23802359.2019.1606679 PMC7721030 33366436

[CIT0017] Nakamura T, Yamada KD, Tomii K, Katoh K. 2018. Parallelization of MAFFT for large-scale multiple sequence alignments. Bioinformatics. 34(14):2490–2492. 10.1093/bioinformatics/bty121 29506019 PMC6041967

[CIT0018] Qi XQ et al. 2021. Characterization of the complete chloroplast genome of *Actinidia hemsleyana*. Mitochondrial DNA Part B. 6(11):3259–3260. 10.1080/23802359.2021.1993100 34693017 PMC8530474

[CIT0019] Ren W et al. 2023. High-quality assembly and comparative analysis of *Actinidia latifolia* and *A. valvata* mitogenomes. Genes (Basel). 14(4):863. 10.3390/genes14040863 37107621 PMC10138172

[CIT0020] Stamatakis A. 2014. RAxML version 8: a tool for phylogenetic analysis and post-analysis of large phylogenies. Bioinformatics. 30(9):1312–1313. 10.1093/bioinformatics/btu033 24451623 PMC3998144

[CIT0021] Tang P, Shen R, He R, Yao X. 2019. The complete chloroplast genome sequence of *Actinidia eriantha*. Mitochondrial DNA B Resour. 4(2):2114–2115. 10.1080/23802359.2019.1623111 33365432 PMC7687386

[CIT0022] Tillich M et al. 2017. GeSeq – versatile and accurate annotation of organelle genomes. Nucleic Acids Res. 45(W1):W6–W11. 10.1093/nar/gkx391 28486635 PMC5570176

[CIT0023] Wang WC, Chen SY, Zhang XZ. 2016. Chloroplast genome evolution in Actinidiaceae: clpP loss, heterogenous divergence and phylogenomic practice. PLoS One. 11(9):e0162324. 10.1371/journal.pone.0162324 27589600 PMC5010200

[CIT0024] Wu HL, Li MM, Wang DY, Liu HH, Xu XT. 2019. The complete chloroplast genome sequence of *Actinidia callosa* var. *henryi*. Mitochondrial DNA Part B. 4(1):652–653. 10.1080/23802359.2018.1561223

[CIT0025] Xu YS et al. 2020. The complete chloroplast genome of *Actinidia rubus* (Actinidiaceae). Mitochondrial DNA B Resour. 5(1):366–367. 10.1080/23802359.2019.1703571 33366559 PMC7748799

[CIT0026] Yang A et al. 2019. The complete chloroplast genome sequence of *Actinidia styracifolia* C. F. Liang. Mitochondrial DNA B Resour. 5(1):90–91. 10.1080/23802359.2019.1698337 33366436 PMC7721030

[CIT0027] Yao X et al. 2015. The first complete chloroplast genome sequences in actinidiaceae: genome structure and comparative analysis. PLoS One. 10(6):e0129347. 10.1371/journal.pone.0129347 26046631 PMC4457681

[CIT0028] Zhang F, Yan Z, Xu Y, Lin H. 2019. The complete chloroplast genome of *Actinidia fulvicoma*. Mitochondrial DNA B Resour. 4(2):4089–4090. 10.1080/23802359.2019.1691949 33366332 PMC7707750

[CIT0029] Zhang J, Liu H. 2019. The complete chloroplast genome sequence of *Actinidia lanceolata*. Mitochond DNA Part B. 4(1):1187–1188. 10.1080/23802359.2019.1591200

[CIT0030] Zhang Q, Wu L, Jiang Q, Huang J, Yao X. 2025. The complete chloroplast genome sequence and phylogenetic analysis of *Actinidia suberifolia* C.Y. Wu (Actinidiaceae). Mitochondrial DNA B Resour. 10(6):465–469. 10.1080/23802359.2025.2503400 40385523 PMC12082740

[CIT0032] Zhou C, Brown M, Blaxter M, McCarthy SA, Durbin R, Darwin Tree of Life Project Consortium. 2025. Oatk: a de novo assembly tool for complex plant organelle genomes. Genome Biol. 26(1):235. 10.1186/s13059-025-03676-6 40775726 PMC12329965

