# Supplementary figures and images for: Characterization of the complete chloroplast genome of Actinidia melliana (Actinidiaceae)
Source: Mitochondrial DNA B Resour. 2026 Jan 1;11(1):222–7. doi: 10.1080/23802359.2025.2611483 (PMC12777877; doi:10.1080/23802359.2025.2611483)

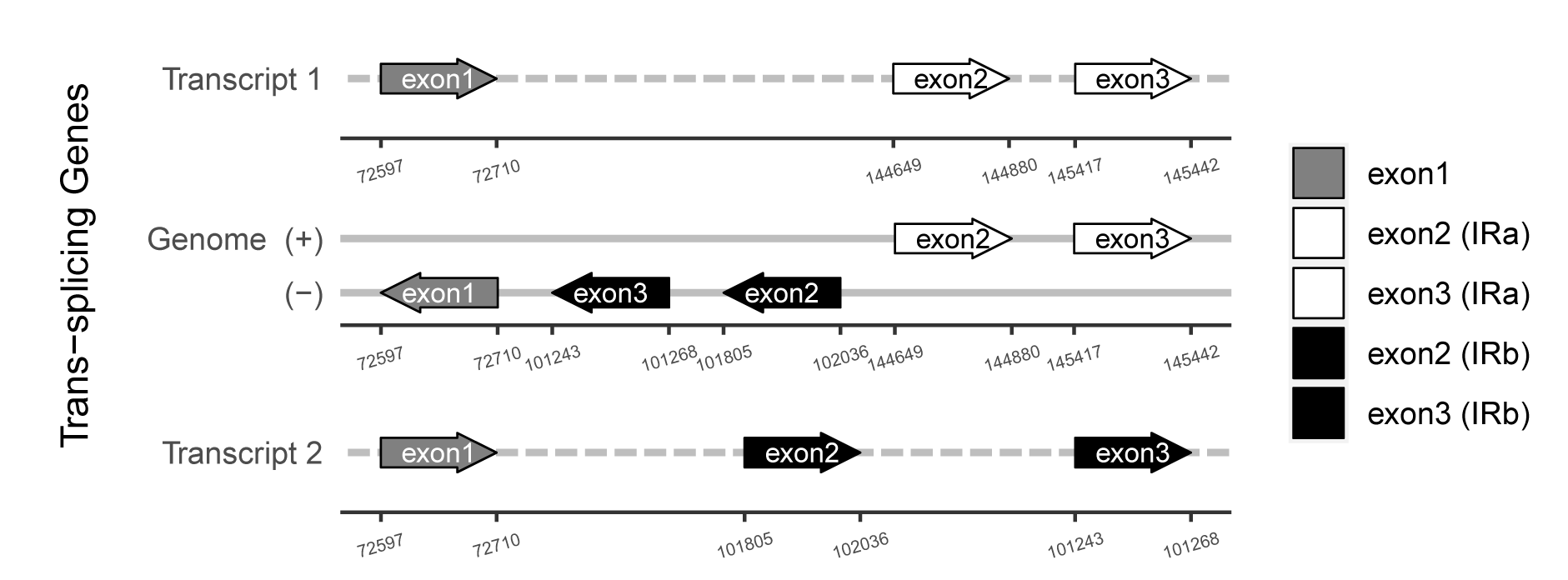

Supplement: FigS3.tif [file TMDN_A_2611483_SM4436.tif]

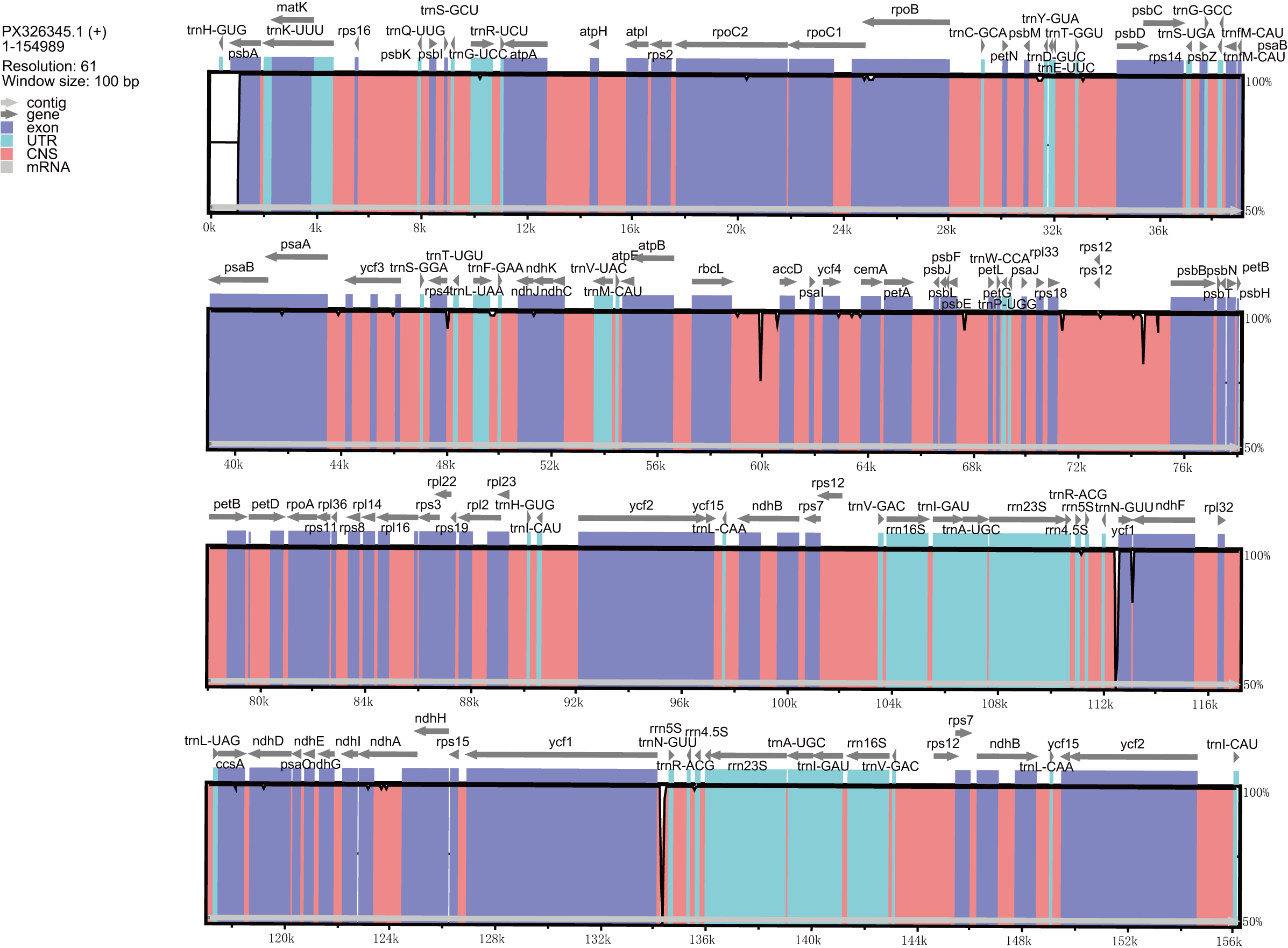

Supplement: FigS4.tif [file TMDN_A_2611483_SM4435.tif]

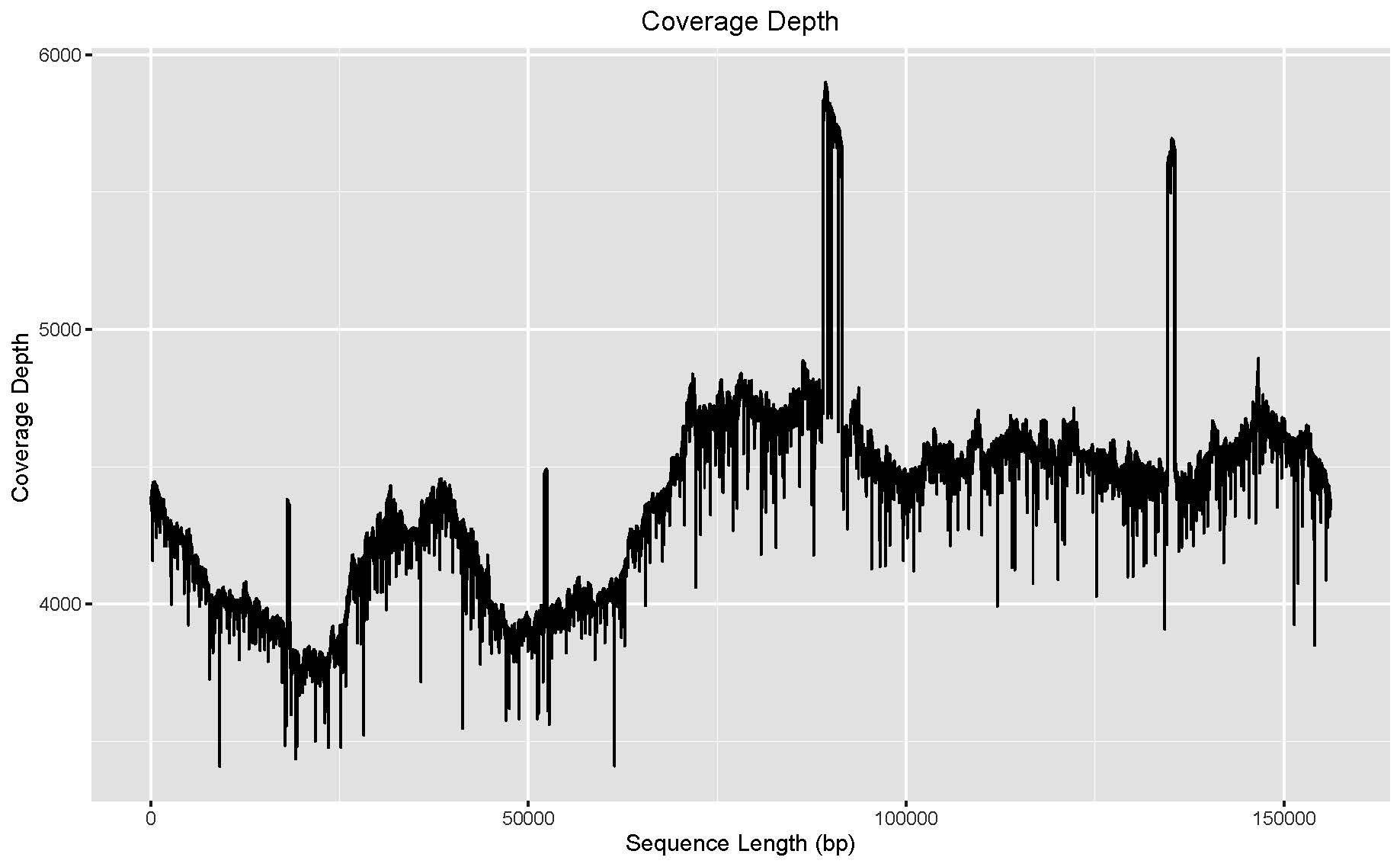

Supplement: FigS1.jpg [file TMDN_A_2611483_SM4434.jpg]

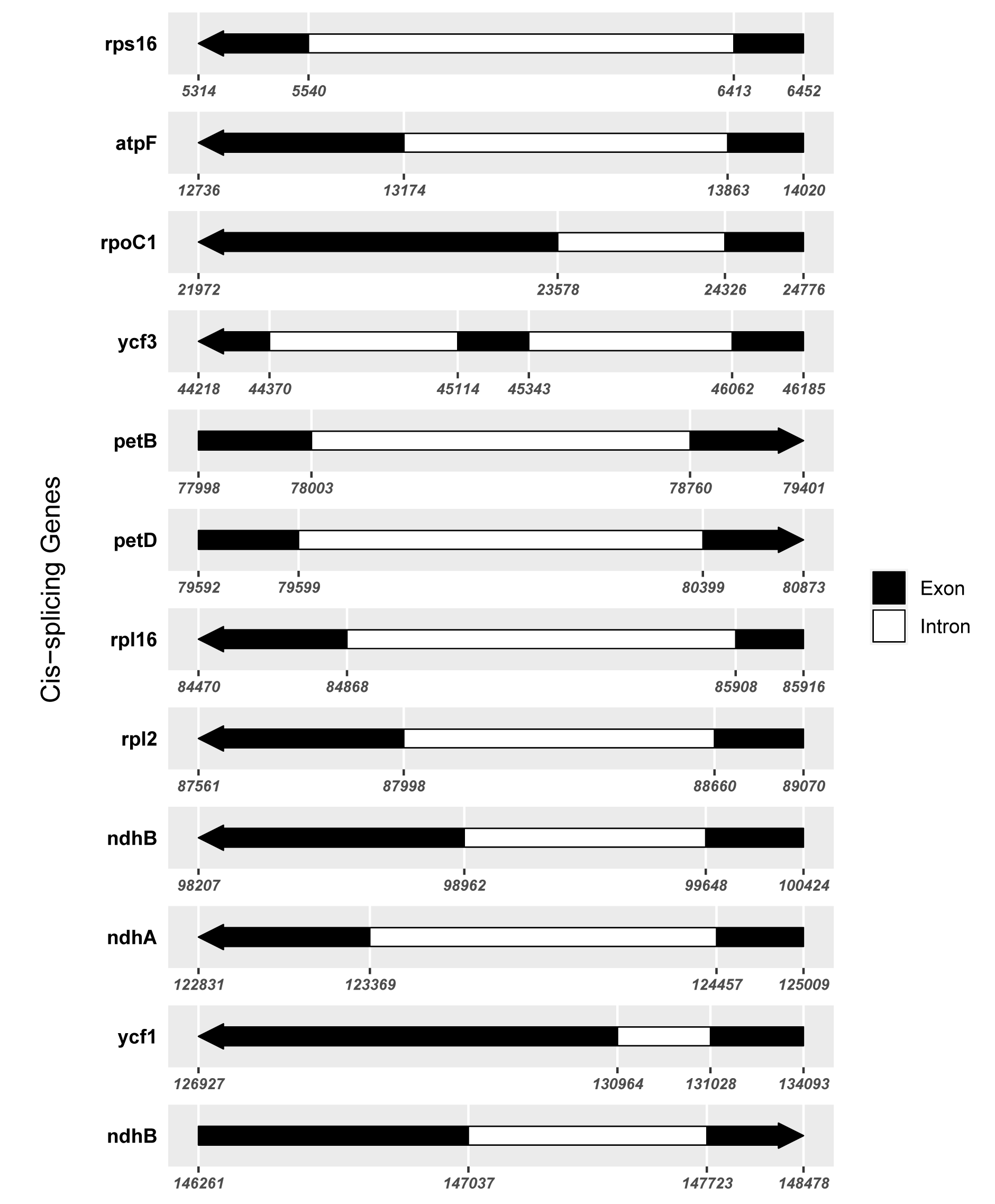

Supplement: figS2.tif [file TMDN_A_2611483_SM4433.tif]
